# Supplementary material for: The Ectonucleotidases CD39 and CD73 and the Purinergic Receptor P2X4 Serve as Prognostic Markers in Non-Small Cell Lung Cancer
Source: Cancers (Basel). 2025 Mar 28;17(7):1142. doi: 10.3390/cancers17071142 (PMC11987875; doi:10.3390/cancers17071142)
Supplement: Supplementary file 1 [file cancers-17-01142-s001.zip › Table S9 Uni- and Multi-variable Cox-Regression of p2x7 Expression.pdf]

Uni- and Multivariable Analysis – P2X7									
	**Absolute**	**Univariable**				**Multivariable**			
**Characteristic**	**N = 138**[1]	**N**	**HR**[2]	**95% CI**[2]	**p-value**	**N**	**HR**[2]	**95% CI**[2]	**p-value**
H-Score: Tumor		138			0.3	137			0.5
high	43 (31%)		—	—			—	—	
low	95 (69%)		1.35	0.79, 2.31			0.77	0.38, 1.54	
H-Score: Stroma		138			0.065	137			0.2
high	37 (27%)		—	—			—	—	
low	101 (73%)		1.71	0.94, 3.13			1.74	0.79, 3.82	
Histology		138			0.6				
AC	74 (54%)		—	—					
SCC	64 (46%)		0.90	0.56, 1.43					
Sex		138			**0.002**	137			**0.016**
Female	43 (31%)		—	—			—	—	
Male	95 (69%)		2.34	1.30, 4.20			2.07	1.11, 3.87	
Age	67 (62, 75)	138	1.00	0.98, 1.03	0.8				
pT		138			**0.005**	137			0.087
pT1	33 (24%)		—	—			—	—	
pT2	65 (47%)		2.24	1.11, 4.55			2.57	1.16, 5.70	
pT3	27 (20%)		3.01	1.39, 6.53			2.22	0.80, 6.11	
pT4	13 (9.4%)		4.11	1.70, 9.94			3.18	1.02, 9.85	
pN		137			**<0.001**	137			**0.049**
pN0	75 (55%)		—	—			—	—	
pN1	33 (24%)		2.46	1.41, 4.30			1.95	1.02, 3.72	
pN2	29 (21%)		2.66	1.50, 4.73			2.02	1.08, 3.80	
Pn		138			0.3				
Pn0	127 (92%)		—	—					
Pn1	11 (8.0%)		1.55	0.74, 3.23					
L		138			**<0.001**				
L0	86 (62%)		—	—					
L1	52 (38%)		2.55	1.60, 4.07					
V		138			**0.004**	137			0.062
V0	120 (87%)		—	—			—	—	
V1	18 (13%)		2.45	1.40, 4.29			1.91	1.00, 3.62	
Grading		138			0.8				
G2	66 (48%)		—	—					
G3	72 (52%)		1.05	0.66, 1.66					

Uni- and Multivariable Analysis – P2X7									
	**Absolute**	**Univariable**				**Multivariable**			
**Characteristic**	**N = 138**[1]	**N**	**HR**[2]	**95% CI**[2]	**p-value**	**N**	**HR**[2]	**95% CI**[2]	**p-value**
Residual Disease		138			**0.001**	137			0.2
R0	125 (91%)		—	—			—	—	
R1	10 (7.2%)		3.92	1.97, 7.78			2.08	0.93, 4.62	
Rx	3 (2.2%)		3.23	1.00, 10.4			1.09	0.28, 4.27	
Pleural Infiltration	52 (38%)	138			**0.017**				
No			—	—					
Yes			1.77	1.11, 2.83					
Metastatic Lymphnodes	0.00 (0.00, 3.00)	137	1.14	1.07, 1.21	**<0.001**				
Tumor Size in cm		138	1.14	1.03, 1.26	**0.013**	137	0.96	0.82, 1.12	0.6
Neoadjuvant Therapy		138			0.2				
No			—	—					
Yes			1.72	0.79, 3.76					
Pack Years		64	1.00	0.99, 1.02	0.7				
SUVmax		137	1.00	0.99, 1.01	0.6				

[1] n (%); Median (Q1, Q3)

[2] HR = Hazard Ratio, CI = Confidence Interval
