# Supplementary material for: The Ectonucleotidases CD39 and CD73 and the Purinergic Receptor P2X4 Serve as Prognostic Markers in Non-Small Cell Lung Cancer
Source: Cancers (Basel). 2025 Mar 28;17(7):1142. doi: 10.3390/cancers17071142 (PMC11987875; doi:10.3390/cancers17071142)
Supplement: Supplementary file 1 [file cancers-17-01142-s001.zip › Table S10 Uni- and Multi-variable Cox-Regression of p2x7 Expression in the SCC Subgroup.pdf]

Uni- and Multivariable Analysis – P2X7 Squamous Cell Carcinoma									
	**Absolute**	**Univariable**				**Multivariable**			
**Characteristic**	**N = 64**[1]	**N**	**HR**[2]	**95% CI**[2]	**p-value**	**N**	**HR**[2]	**95% CI**[2]	**p-value**
H-Score: Tumor		64			0.5	64			0.6
high	16 (25%)		—	—			—	—	
low	48 (75%)		1.36	0.56, 3.33			0.69	0.16, 2.98	
H-Score: Stroma		64			0.4	64			0.7
high	19 (30%)		—	—			—	—	
low	45 (70%)		1.46	0.63, 3.38			1.26	0.31, 5.08	
Sex		64			**0.009**	64			0.070
Female	15 (23%)		—	—			—	—	
Male	49 (77%)		3.80	1.16, 12.5			2.96	0.80, 11.0	
Age	70 (63, 75)	64	0.99	0.95, 1.03	0.5				
pT		64			**0.003**	64			0.055
pT1	13 (20%)		—	—			—	—	
pT2	28 (44%)		1.97	0.63, 6.13			1.92	0.49, 7.52	
pT3	14 (22%)		2.82	0.83, 9.53			2.34	0.38, 14.2	
pT4	9 (14%)		8.82	2.59, 30.0			7.41	1.34, 41.0	
pN		64			0.3	64			0.7
pN0	31 (48%)		—	—			—	—	
pN1	19 (30%)		1.44	0.63, 3.26			1.33	0.50, 3.51	
pN2	14 (22%)		1.93	0.83, 4.48			1.53	0.58, 4.00	
Pn		64			0.3				
Pn0	56 (88%)		—	—					
Pn1	8 (13%)		1.59	0.65, 3.86					
L		64			0.093				
L0	37 (58%)		—	—					
L1	27 (42%)		1.80	0.91, 3.59					
V		64			**0.014**	64			0.2
V0	53 (83%)		—	—			—	—	
V1	11 (17%)		2.77	1.31, 5.87			1.78	0.71, 4.44	
Grading		64			0.5				
G2	29 (45%)		—	—					
G3	35 (55%)		0.81	0.41, 1.61					
Residual Disease		64			**0.026**				
R0	56 (88%)		—	—					
R1	5 (7.8%)		3.28	1.22, 8.79					

Uni- and Multivariable Analysis – P2X7 Squamous Cell Carcinoma									
	Absolute	Univariable				Multivariable			
**Characteristic**	**N = 64[1]**	**N**	**HR[2]**	**95% CI[2]**	**p-value**	**N**	**HR[2]**	**95% CI[2]**	**p-value**
Rx	3 (4.7%)		4.09	1.19, 14.1					
Pleural Infiltration	24 (38%)	64			0.053				
No			—	—					
Yes			2.02	1.00, 4.06					
Metastatic Lymphnodes	1.00 (0.00, 3.00)	64	1.09	0.96, 1.24	0.2				
Tumor Size in cm		64	1.16	1.00, 1.35	**0.045**	64	0.94	0.73, 1.21	0.6
Neoadjuvant Therapy		64			0.6				
No			—	—					
Yes			1.58	0.38, 6.65					
Pack Years		33	0.99	0.97, 1.02	0.4				
SUVmax		64	1.01	1.00, 1.02	0.2				

[1]n (%); Median (Q1, Q3)

[2]HR = Hazard Ratio, CI = Confidence Interval
