# Supplementary material for: The Ectonucleotidases CD39 and CD73 and the Purinergic Receptor P2X4 Serve as Prognostic Markers in Non-Small Cell Lung Cancer
Source: Cancers (Basel). 2025 Mar 28;17(7):1142. doi: 10.3390/cancers17071142 (PMC11987875; doi:10.3390/cancers17071142)
Supplement: Supplementary file 1 [file cancers-17-01142-s001.zip › Table S11 Uni- and Multi-variable Cox-Regression of p2x7 Expression in the ADC Subgroup.pdf]

| Uni- and Multivariable Analysis – P2X7 Adenocarcinoma | | | | | | | | | |
|---|---|---|---|---|---|---|---|---|---|
| | **Absolute** | **Univariable** | | | | **Multivariable** | | | |
| **Characteristic** | **N = 74**[1] | **N** | **HR**[2] | **95% CI**[2] | **p-value** | **N** | **HR**[2] | **95% CI**[2] | **p-value** |
| H-Score: Tumor | | 74 | | | 0.3 | 73 | | | 0.6 |
| high | 27 (36%) | | — | — | | | — | — | |
| low | 47 (64%) | | 1.40 | 0.71, 2.76 | | | 0.78 | 0.31, 1.99 | |
| H-Score: Stroma | | 74 | | | 0.10 | 73 | | | 0.12 |
| high | 18 (24%) | | — | — | | | — | — | |
| low | 56 (76%) | | 1.98 | 0.83, 4.72 | | | 2.40 | 0.76, 7.54 | |
| Sex | | 74 | | | **0.050** | 73 | | | 0.12 |
| Female | 28 (38%) | | — | — | | | — | — | |
| Male | 46 (62%) | | 1.96 | 0.97, 3.94 | | | 1.91 | 0.83, 4.39 | |
| Age | 67 (59, 74) | 74 | 1.01 | 0.98, 1.05 | 0.4 | | | | |
| pT | | 74 | | | **0.036** | 73 | | | 0.2 |
| pT1 | 20 (27%) | | — | — | | | — | — | |
| pT2 | 37 (50%) | | 2.52 | 1.02, 6.23 | | | 2.34 | 0.78, 6.99 | |
| pT3 | 13 (18%) | | 3.61 | 1.31, 9.95 | | | 1.85 | 0.48, 7.14 | |
| pT4 | 4 (5.4%) | | 0.86 | 0.10, 7.14 | | | 0.57 | 0.06, 5.65 | |
| pN | | 73 | | | **<0.001** | 73 | | | **0.008** |
| pN0 | 44 (60%) | | — | — | | | — | — | |
| pN1 | 14 (19%) | | 4.65 | 2.16, 10.0 | | | 3.83 | 1.41, 10.4 | |
| pN2 | 15 (21%) | | 3.53 | 1.61, 7.73 | | | 3.33 | 1.36, 8.16 | |
| Pn | | 74 | | | 0.5 | | | | |
| Pn0 | 71 (96%) | | — | — | | | | | |
| Pn1 | 3 (4.1%) | | 1.65 | 0.40, 6.87 | | | | | |
| L | | 74 | | | **<0.001** | | | | |
| L0 | 49 (66%) | | — | — | | | | | |
| L1 | 25 (34%) | | 3.79 | 1.99, 7.21 | | | | | |
| V | | 74 | | | 0.11 | 73 | | | 0.7 |
| V0 | 67 (91%) | | — | — | | | — | — | |
| V1 | 7 (9.5%) | | 2.19 | 0.91, 5.24 | | | 1.30 | 0.42, 4.00 | |
| Grading | | 74 | | | 0.4 | | | | |
| G2 | 37 (50%) | | — | — | | | | | |
| G3 | 37 (50%) | | 1.34 | 0.71, 2.52 | | | | | |
| Residual Disease | | 74 | | | **0.003** | 73 | | | 0.12 |
| R0 | 69 (93%) | | — | — | | | — | — | |
| R1 | 5 (6.8%) | | 6.07 | 2.27, 16.2 | | | 2.82 | 0.82, 9.70 | |

| Uni- and Multivariable Analysis – P2X7 Adenocarcinoma | | | | | | | | | |
|---|---|---|---|---|---|---|---|---|---|
| | **Absolute** | **Univariable** | | | | **Multivariable** | | | |
| **Characteristic** | **N = 74**[1] | **N** | **HR**[2] | **95% CI**[2] | **p-value** | **N** | **HR**[2] | **95% CI**[2] | **p-value** |
| Pleural Infiltration | 28 (38%) | 74 | | | 0.11 | | | | |
| No | | | — | — | | | | | |
| Yes | | | 1.67 | 0.89, 3.15 | | | | | |
| Metastatic Lymphnodes | 0.00 (0.00, 2.00) | 73 | 1.15 | 1.08, 1.24 | **<0.001** | | | | |
| Tumor Size in cm | | 74 | 1.16 | 1.00, 1.34 | 0.067 | 73 | 1.14 | 0.92, 1.42 | 0.2 |
| Neoadjuvant Therapy | | 74 | | | 0.3 | | | | |
| No | | | — | — | | | | | |
| Yes | | | 1.74 | 0.68, 4.44 | | | | | |
| Pack Years | | 31 | 1.01 | 0.99, 1.03 | 0.3 | | | | |
| SUVmax | | 73 | 1.00 | 0.99, 1.01 | >0.9 | | | | |

[1]n (%); Median (Q1, Q3)

[2]HR = Hazard Ratio, CI = Confidence Interval
