# Supplementary material for: The Ectonucleotidases CD39 and CD73 and the Purinergic Receptor P2X4 Serve as Prognostic Markers in Non-Small Cell Lung Cancer
Source: Cancers (Basel). 2025 Mar 28;17(7):1142. doi: 10.3390/cancers17071142 (PMC11987875; doi:10.3390/cancers17071142)
Supplement: Supplementary file 1 [file cancers-17-01142-s001.zip › Table S12 Uni- and Multi-variable Cox-Regression of p2x4 Expression.pdf]

Uni- and Multivariable Analysis – P2X4									
	Absolute	Univariable				Multivariable			
Characteristic	N = 136[1]	N	HR[2]	95% CI[2]	p-value	N	HR[2]	95% CI[2]	p-value
H-Score: Tumor		136			0.2	135			**0.039**
high	77 (57%)		—	—			—	—	
low	59 (43%)		1.32	0.83, 2.10			1.95	1.04, 3.65	
H-Score: Stroma		136			0.2	135			**0.004**
high	93 (68%)		—	—			—	—	
low	43 (32%)		0.72	0.42, 1.22			0.39	0.20, 0.75	
Histology		136			0.8				
AC	73 (54%)		—	—					
SCC	63 (46%)		0.93	0.59, 1.49					
Sex		136			**0.002**	135			**0.013**
Female	43 (32%)		—	—			—	—	
Male	93 (68%)		2.35	1.31, 4.22			2.13	1.14, 3.98	
Age	67 (62, 75)	136	1.00	0.98, 1.03	0.8				
pT		136			**0.004**	135			0.074
pT1	33 (24%)		—	—			—	—	
pT2	64 (47%)		2.19	1.08, 4.46			2.37	1.07, 5.28	
pT3	26 (19%)		3.20	1.47, 6.93			2.54	0.92, 7.02	
pT4	13 (9.6%)		4.13	1.71, 9.99			4.35	1.40, 13.6	
pN		135			**<0.001**	135			**0.039**
pN0	74 (55%)		—	—			—	—	
pN1	32 (24%)		2.69	1.53, 4.73			2.16	1.18, 3.97	
pN2	29 (21%)		2.76	1.55, 4.92			1.75	0.91, 3.38	
Pn		136			0.14				
Pn0	126 (93%)		—	—					
Pn1	10 (7.4%)		1.82	0.87, 3.81					
L		136			**<0.001**				
L0	84 (62%)		—	—					
L1	52 (38%)		2.59	1.62, 4.14					
V		136			**0.004**	135			**0.046**
V0	118 (87%)		—	—			—	—	
V1	18 (13%)		2.47	1.41, 4.33			1.87	1.04, 3.36	
Grading		136			0.7				
G2	65 (48%)		—	—					
G3	71 (52%)		1.10	0.69, 1.75					

Uni- and Multivariable Analysis – P2X4

	Absolute	Univariable				Multivariable			
**Characteristic**	**N = 136**[1]	**N**	**HR**[2]	**95% CI**[2]	**p-value**	**N**	**HR**[2]	**95% CI**[2]	**p-value**
Residual Disease		136			**0.003**				
R0	124 (91%)		—	—					
R1	9 (6.6%)		3.71	1.81, 7.59					
Rx	3 (2.2%)		3.21	1.0, 10.3					
Pleural Infiltration	51 (38%)	136			**0.027**				
No			—	—					
Yes			1.71	1.07, 2.73					
Metastatic Lymphnodes	0.00 (0.00, 3.00)	135	1.14	1.07, 1.22	**<0.001**				
Tumor Size in cm		136	1.16	1.05, 1.28	**0.006**	135	1.00	0.85, 1.17	>0.9
Neoadjuvant Therapy		136			0.2				
No			—	—					
Yes			1.72	0.79, 3.76					
Pack Years		62	1.00	0.99, 1.02	>0.9				
SUVmax		135	1.00	0.99, 1.01	0.6				

[1]n (%); Median (Q1, Q3)

[2]HR = Hazard Ratio, CI = Confidence Interval
