# Supplementary material for: The Ectonucleotidases CD39 and CD73 and the Purinergic Receptor P2X4 Serve as Prognostic Markers in Non-Small Cell Lung Cancer
Source: Cancers (Basel). 2025 Mar 28;17(7):1142. doi: 10.3390/cancers17071142 (PMC11987875; doi:10.3390/cancers17071142)
Supplement: Supplementary file 1 [file cancers-17-01142-s001.zip › Table S14 Uni- and Multi-variable Cox-Regression of P2X4 Expression in the ADC Subgroup.pdf]

Uni- and Multivariable Analysis – P2X4 Adenocarcinoma									
	**Absolute**	**Univariable**				**Multivariable**			
**Characteristic**	**N = 73**[1]	**N**	**HR**[2]	**95% CI**[2]	**p-value**	**N**	**HR**[2]	**95% CI**[2]	**p-value**
H-Score: Tumor		73			0.13	72			**0.018**
high	47 (64%)		—	—			—	—	
low	26 (36%)		1.66	0.88, 3.16			2.92	1.23, 6.89	
H-Score: Stroma		73			0.8	72			0.087
high	57 (78%)		—	—			—	—	
low	16 (22%)		0.89	0.41, 1.94			0.41	0.15, 1.16	
Sex		73			0.062	72			**0.033**
Female	28 (38%)		—	—			—	—	
Male	45 (62%)		1.90	0.94, 3.84			2.36	1.03, 5.41	
Age	67 (59, 74)	73	1.02	0.98, 1.05	0.4				
pT		73			**0.041**	72			**0.016**
pT1	20 (27%)		—	—			—	—	
pT2	36 (49%)		2.44	0.98, 6.06			3.85	1.36, 10.9	
pT3	13 (18%)		3.61	1.31, 9.94			4.49	1.31, 15.4	
pT4	4 (5.5%)		0.86	0.10, 7.16			1.03	0.11, 9.90	
pN		72			**<0.001**	72			**0.009**
pN0	43 (60%)		—	—			—	—	
pN1	14 (19%)		4.99	2.29, 10.9			3.38	1.37, 8.33	
pN2	15 (21%)		3.78	1.70, 8.39			3.18	1.32, 7.66	
Pn		73			0.5				
Pn0	70 (96%)		—	—					
Pn1	3 (4.1%)		1.69	0.41, 7.04					
L		73			**<0.001**				
L0	48 (66%)		—	—					
L1	25 (34%)		4.01	2.09, 7.70					
V		73			0.10	72			0.2
V0	66 (90%)		—	—			—	—	
V1	7 (9.6%)		2.25	0.94, 5.41			1.94	0.71, 5.32	
Grading		73			0.3				
G2	36 (49%)		—	—					
G3	37 (51%)		1.41	0.74, 2.67					
Residual Disease		73			**0.008**				
R0	69 (95%)		—	—					
R1	4 (5.5%)		5.81	1.99, 16.9					

Uni- and Multivariable Analysis – P2X4 Adenocarcinoma									
	Absolute	Univariable				Multivariable			
Characteristic	N = 73[1]	N	HR[2]	95% CI[2]	p-value	N	HR[2]	95% CI[2]	p-value
Pleural Infiltration	27 (37%)	73			0.2				
No			—	—					
Yes			1.61	0.85, 3.05					
Metastatic Lymphnodes	0.00 (0.00, 2.00)	72	1.16	1.08, 1.24	**<0.001**				
Tumor Size in cm		73	1.17	1.00, 1.35	0.055				
Neoadjuvant Therapy		73			0.3				
No			—	—					
Yes			1.78	0.69, 4.55					
Pack Years		30	1.01	0.99, 1.03	0.4				
SUVmax		72	1.00	0.99, 1.01	>0.9				

[1]n (%); Median (Q1, Q3)

[2]HR = Hazard Ratio, CI = Confidence Interval
