# Supplementary material for: The Ectonucleotidases CD39 and CD73 and the Purinergic Receptor P2X4 Serve as Prognostic Markers in Non-Small Cell Lung Cancer
Source: Cancers (Basel). 2025 Mar 28;17(7):1142. doi: 10.3390/cancers17071142 (PMC11987875; doi:10.3390/cancers17071142)
Supplement: Supplementary file 1 [file cancers-17-01142-s001.zip › Table S1 Clinicopathological Parameter.pdf]

Clinicopathological Parameters	
**Characteristic**	**N = 139**[1]
Histology	
ADC	75 (54%)
SCC	64 (46%)
Sex	
Female	43 (31%)
Male	96 (69%)
Age	67 (10(67, 35 - 90)
pT	
pT1	33 (24%)
pT2	66 (47%)
pT3	27 (19%)
pT4	13 (9.4%)
pN	
pN0	75 (54%)
pN1	34 (25%)
pN2	29 (21%)
Unknown	1
Pn	
Pn0	128 (92%)
Pn1	11 (7.9%)
L	
L0	86 (62%)
L1	53 (38%)
V	
V0	121 (87%)
V1	18 (13%)
Grading	
G2	66 (47%)
G3	73 (53%)
Residual Disease	
R0	126 (91%)
R1	10 (7.2%)
Rx	3 (2.2%)
Tumor Size in cm	4.38 (2.22(4.20, 0.70 - 10.50)
Neoadjuvant Therapy	10 (7.2%)
Pack Years	45 (23(40, 2 - 100)
Unknown	74
SUVmax	20 (26(12, 2 - 99)
Unknown	1

Clinicopathological Parameters	
**Characteristic**	**N = 139**[1]
Relapse	46 (34%)
Unknown	2
Distant Metastasis	16 (12%)
Pleural Infiltration	52 (37%)
Metastatic Lymphnodes	1.72 (2.76(0.00, 0.00 - 15.00)
Unknown	1
Deceased	49 (35%)
Progression-free Survival (months)	44 (29(51, 2 - 94)
Progression-free survival	73 (53%)

[1]n (%); Mean (SD(Median, Min - Max)
