# Supplementary material for: The Ectonucleotidases CD39 and CD73 and the Purinergic Receptor P2X4 Serve as Prognostic Markers in Non-Small Cell Lung Cancer
Source: Cancers (Basel). 2025 Mar 28;17(7):1142. doi: 10.3390/cancers17071142 (PMC11987875; doi:10.3390/cancers17071142)
Supplement: Supplementary file 1 [file cancers-17-01142-s001.zip › Table S2 Antibody Expression.pdf]

Antibody Expression	
**Characteristic**	**N = 139**[1]
CD39 - H-Score: Tumor	25 (43(7, 0 - 246)
CD39 - Relative Expression: Tumor	20 (28(6, 0 - 100)
CD39 - H-Score: Stroma	124 (50(121, 14 - 250)
CD39 - Relative Expression: Stroma	75 (21(81, 13 - 100)
CD73 - H-Score: Tumor	39 (61(10, 0 - 228)
CD73 - Relative Expression: Tumor	23 (30(9, 0 - 96)
CD73 - H-Score: Stroma	39 (42(22, 0 - 178)
CD73 - Relative Expression: Stroma	28 (25(18, 0 - 93)
P2X7 - H-Score: Tumor	177 (50(179, 82 - 283)
Unknown	1
P2X7 - Relative Expression: Tumor	97.7 (4.7(100.0, 76.3 - 100.0)
Unknown	1
P2X7 - H-Score: Stroma	90 (21(97, 36 - 125)
Unknown	1
P2X7 - Relative Expression: Stroma	84 (19(95, 33 - 100)
Unknown	1
P2X4 - H-Score: Tumor	48 (54(19, 0 - 223)
Unknown	3
P2X4 - Relative Expression: Tumor	32 (33(16, 0 - 98)
Unknown	3
P2X4 - H-Score: Stroma	21 (25(7, 0 - 96)
Unknown	3
P2X4 - Relative Expression: Stroma	17 (20(6, 0 - 80)
Unknown	3

[1]Mean (SD(Median, Min - Max)
