# Supplementary material for: The Ectonucleotidases CD39 and CD73 and the Purinergic Receptor P2X4 Serve as Prognostic Markers in Non-Small Cell Lung Cancer
Source: Cancers (Basel). 2025 Mar 28;17(7):1142. doi: 10.3390/cancers17071142 (PMC11987875; doi:10.3390/cancers17071142)
Supplement: Supplementary file 1 [file cancers-17-01142-s001.zip › Table S3 Uni- and Multi-variable Cox-Regression of CD39 Expression.pdf]

Uni- and Multivariable Analysis - CD39									
	Absolute		Univariable				Multivariable		
Characteristic	N = 139[1]	N	HR[2]	95% CI[2]	p-value	N	HR[2]	95% CI[2]	p-value
H-Score: Tumor		139			**0.007**	138			0.4
high	83 (60%)		—	—			—	—	
low	56 (40%)		1.89	1.19, 2.99			1.25	0.74, 2.09	
H-Score: Stroma		139			**0.004**	138			**0.004**
high	41 (29%)		—	—			—	—	
low	98 (71%)		2.20	1.23, 3.94			2.49	1.30, 4.76	
Histology		139			0.6				
AC	75 (54%)		—	—					
SCC	64 (46%)		0.89	0.56, 1.42					
Sex		139			**0.002**	138			**0.011**
Female	43 (31%)		—	—			—	—	
Male	96 (69%)		2.32	1.29, 4.16			2.12	1.15, 3.92	
Age	67 (62, 75)	139	1.00	0.98, 1.03	0.8				
pT		139			**0.004**	138			0.082
pT1	33 (24%)		—	—			—	—	
pT2	66 (47%)		2.26	1.12, 4.56			2.67	1.20, 5.93	
pT3	27 (19%)		3.02	1.39, 6.56			2.59	0.95, 7.08	
pT4	13 (9.4%)		4.18	1.73, 10.1			3.04	1.02, 9.02	
pN		138			**<0.001**	138			0.064
pN0	75 (54%)		—	—			—	—	
pN1	34 (25%)		2.45	1.41, 4.25			1.65	0.90, 3.05	
pN2	29 (21%)		2.69	1.51, 4.78			2.01	1.09, 3.70	
Pn		139			0.3				
Pn0	128 (92%)		—	—					
Pn1	11 (7.9%)		1.54	0.74, 3.21					
L		139			**<0.001**				
L0	86 (62%)		—	—					
L1	53 (38%)		2.53	1.59, 4.03					
V		139			**0.004**	138			**0.009**
V0	121 (87%)		—	—			—	—	
V1	18 (13%)		2.45	1.40, 4.28			2.40	1.29, 4.47	
Grading		139			0.8				
G2	66 (47%)		—	—					
G3	73 (53%)		1.06	0.67, 1.68					

Uni- and Multivariable Analysis - CD39									
	**Absolute**	**Univariable**				**Multivariable**			
**Characteristic**	**N = 139**[1]	**N**	**HR**[2]	**95% CI**[2]	**p-value**	**N**	**HR**[2]	**95% CI**[2]	**p-value**
Residual Disease		139			**0.001**				
R0	126 (91%)		—	—					
R1	10 (7.2%)		3.96	1.99, 7.86					
Rx	3 (2.2%)		3.26	1.01, 10.5					
Pleural Infiltration	52 (37%)	139			**0.018**				
No			—	—					
Yes			1.76	1.11, 2.80					
Metastatic Lymphnodes	0.00 (0.00, 3.00)	138	1.14	1.07, 1.21	**<0.001**				
Tumor Size in cm		139	1.14	1.03, 1.26	**0.013**	138	0.99	0.85, 1.16	0.9
Neoadjuvant Therapy		139			0.2				
No			—	—					
Yes			1.74	0.80, 3.80					
Pack Years		65	1.00	0.99, 1.02	0.7				
SUVmax		138	1.00	0.99, 1.01	0.6				

[1]n (%); Median (Q1, Q3)

[2]HR = Hazard Ratio, CI = Confidence Interval
