# Supplementary material for: The Ectonucleotidases CD39 and CD73 and the Purinergic Receptor P2X4 Serve as Prognostic Markers in Non-Small Cell Lung Cancer
Source: Cancers (Basel). 2025 Mar 28;17(7):1142. doi: 10.3390/cancers17071142 (PMC11987875; doi:10.3390/cancers17071142)
Supplement: Supplementary file 1 [file cancers-17-01142-s001.zip › Table S4 Uni- and Multi-variable Cox-Regression of CD39 Expression in the SCC subgroup.pdf]

Uni- and Multivariable Analysis - CD39 Squamous Cell Carcinoma									
	**Absolute**	**Univariable**				**Multivariable**			
**Characteristic**	**N = 64**[1]	**N**	**HR**[2]	**95% CI**[2]	**p-value**	**N**	**HR**[2]	**95% CI**[2]	**p-value**
H-Score: Tumor		64			0.11	64			0.5
high	34 (53%)		—	—			—	—	
low	30 (47%)		1.77	0.88, 3.54			1.29	0.57, 2.94	
H-Score: Stroma		64			0.086	64			0.3
high	28 (44%)		—	—			—	—	
low	36 (56%)		1.87	0.90, 3.89			1.56	0.63, 3.86	
Sex		64			**0.009**	64			0.067
Female	15 (23%)		—	—			—	—	
Male	49 (77%)		3.80	1.16, 12.5			2.81	0.81, 9.67	
Age	70 (63, 75)	64	0.99	0.95, 1.03	0.5				
pT		64			**0.003**	64			0.2
pT1	13 (20%)		—	—			—	—	
pT2	28 (44%)		1.97	0.63, 6.13			2.05	0.54, 7.78	
pT3	14 (22%)		2.82	0.83, 9.53			2.14	0.35, 13.0	
pT4	9 (14%)		8.82	2.59, 30.0			5.29	0.92, 30.4	
pN		64			0.3	64			0.6
pN0	31 (48%)		—	—			—	—	
pN1	19 (30%)		1.44	0.63, 3.26			1.30	0.51, 3.30	
pN2	14 (22%)		1.93	0.83, 4.48			1.55	0.62, 3.91	
Pn		64			0.3				
Pn0	56 (88%)		—	—					
Pn1	8 (13%)		1.59	0.65, 3.86					
L		64			0.093				
L0	37 (58%)		—	—					
L1	27 (42%)		1.80	0.91, 3.59					
V		64			**0.014**	64			0.2
V0	53 (83%)		—	—			—	—	
V1	11 (17%)		2.77	1.31, 5.87			1.93	0.76, 4.91	
Grading		64			0.5				
G2	29 (45%)		—	—					
G3	35 (55%)		0.81	0.41, 1.61					
Residual Disease		64			**0.026**				
R0	56 (88%)		—	—					
R1	5 (7.8%)		3.28	1.22, 8.79					

Uni- and Multivariable Analysis - CD39 Squamous Cell Carcinoma									
	**Absolute**	**Univariable**				**Multivariable**			
**Characteristic**	**N = 64**[1]	**N**	**HR**[2]	**95% CI**[2]	**p-value**	**N**	**HR**[2]	**95% CI**[2]	**p-value**
Rx	3 (4.7%)		4.09	1.19, 14.1					
Pleural Infiltration	24 (38%)	64			0.053				
No			—	—					
Yes			2.02	1.00, 4.06					
Metastatic Lymphnodes	1.00 (0.00, 3.00)	64	1.09	0.96, 1.24	0.2				
Tumor Size in cm		64	1.16	1.00, 1.35	**0.045**	64	0.98	0.76, 1.26	0.9
Neoadjuvant Therapy		64			0.6				
No			—	—					
Yes			1.58	0.38, 6.65					
Pack Years		33	0.99	0.97, 1.02	0.4				
SUVmax		64	1.01	1.00, 1.02	0.2				

[1]n (%); Median (Q1, Q3)

[2]HR = Hazard Ratio, CI = Confidence Interval
