# Supplementary material for: The Ectonucleotidases CD39 and CD73 and the Purinergic Receptor P2X4 Serve as Prognostic Markers in Non-Small Cell Lung Cancer
Source: Cancers (Basel). 2025 Mar 28;17(7):1142. doi: 10.3390/cancers17071142 (PMC11987875; doi:10.3390/cancers17071142)
Supplement: Supplementary file 1 [file cancers-17-01142-s001.zip › Table S5 Uni- and Multi-variable Cox-Regression of CD39 Expression in the ADC subgroup.pdf]

Uni- and Multivariable Analysis - CD39 Adenocarcinoma									
	Absolute	Univariable				Multivariable			
Characteristic	N = 75[1]	N	HR[2]	95% CI[2]	p-value	N	HR[2]	95% CI[2]	p-value
H-Score: Tumor		75			**0.014**	74			0.6
high	49 (65%)		—	—			—	—	
low	26 (35%)		2.23	1.19, 4.16			1.24	0.55, 2.81	
H-Score: Stroma		75			**0.011**	74			0.051
high	13 (17%)		—	—			—	—	
low	62 (83%)		3.58	1.10, 11.6			3.16	0.88, 11.4	
Sex		75			**0.049**	74			0.2
Female	28 (37%)		—	—			—	—	
Male	47 (63%)		1.96	0.98, 3.92			1.69	0.74, 3.88	
Age	67 (59, 74)	75	1.02	0.98, 1.05	0.4				
pT		75			**0.038**	74			0.069
pT1	20 (27%)		—	—			—	—	
pT2	38 (51%)		2.51	1.02, 6.17			3.24	1.06, 9.91	
pT3	13 (17%)		3.60	1.31, 9.92			2.90	0.81, 10.4	
pT4	4 (5.3%)		0.88	0.11, 7.29			0.69	0.07, 6.66	
pN		74			**<0.001**	74			0.055
pN0	44 (59%)		—	—			—	—	
pN1	15 (20%)		4.32	2.05, 9.12			1.83	0.67, 5.00	
pN2	15 (20%)		3.64	1.66, 8.01			3.23	1.27, 8.20	
Pn		75			0.5				
Pn0	72 (96%)		—	—					
Pn1	3 (4.0%)		1.69	0.41, 7.01					
L		75			**<0.001**				
L0	49 (65%)		—	—					
L1	26 (35%)		3.66	1.94, 6.87					
V		75			0.10	74			0.2
V0	68 (91%)		—	—			—	—	
V1	7 (9.3%)		2.23	0.93, 5.35			2.05	0.72, 5.84	
Grading		75			0.3				
G2	37 (49%)		—	—					
G3	38 (51%)		1.35	0.73, 2.53					
Residual Disease		75			**0.003**	74			0.2
R0	70 (93%)		—	—			—	—	
R1	5 (6.7%)		6.17	2.31, 16.5			2.37	0.71, 7.94	

Uni- and Multivariable Analysis - CD39 Adenocarcinoma									
	**Absolute**	**Univariable**				**Multivariable**			
**Characteristic**	**N = 75**[1]	**N**	**HR**[2]	**95% CI**[2]	**p-value**	**N**	**HR**[2]	**95% CI**[2]	**p-value**
Pleural Infiltration	28 (37%)	75			0.13				
No			—	—					
Yes			1.63	0.87, 3.04					
Metastatic Lymphnodes	0.00 (0.00, 2.00)	74	1.15	1.08, 1.24	**<0.001**				
Tumor Size in cm		75	1.15	1.00, 1.34	0.066	74	1.07	0.85, 1.36	0.6
Neoadjuvant Therapy		75			0.3				
No			—	—					
Yes			1.77	0.69, 4.54					
Pack Years		32	1.01	0.99, 1.03	0.2				
SUVmax		74	1.00	0.99, 1.01	>0.9				

[1]n (%); Median (Q1, Q3)

[2]HR = Hazard Ratio, CI = Confidence Interval
