# Supplementary material for: The Ectonucleotidases CD39 and CD73 and the Purinergic Receptor P2X4 Serve as Prognostic Markers in Non-Small Cell Lung Cancer
Source: Cancers (Basel). 2025 Mar 28;17(7):1142. doi: 10.3390/cancers17071142 (PMC11987875; doi:10.3390/cancers17071142)
Supplement: Supplementary file 1 [file cancers-17-01142-s001.zip › Table S8 Uni- and Multi-variable Cox-Regression of CD73 Expression in the ADC Subgroup.pdf]

Uni- and Multivariable Analysis – CD73 Adenocarcinoma									
	**Absolute**	**Univariable**				**Multivariable**			
**Characteristic**	**N = 75**[1]	**N**	**HR**[2]	**95% CI**[2]	**p-value**	**N**	**HR**[2]	**95% CI**[2]	**p-value**
H-Score: Tumor		75			0.11	74			**0.014**
high	47 (63%)		—	—			—	—	
low	28 (37%)		0.58	0.30, 1.15			0.40	0.19, 0.85	
H-Score: Stroma		75			>0.9	74			0.3
high	2 (2.7%)		—	—			—	—	
low	73 (97%)		0.95	0.13, 6.93			2.68	0.32, 22.6	
Sex		75			**0.049**	74			0.3
Female	28 (37%)		—	—			—	—	
Male	47 (63%)		1.96	0.98, 3.92			1.56	0.70, 3.47	
Age	67 (59, 74)	75	1.02	0.98, 1.05	0.4				
pT		75			**0.038**	74			0.2
pT1	20 (27%)		—	—			—	—	
pT2	38 (51%)		2.51	1.02, 6.17			2.49	0.86, 7.21	
pT3	13 (17%)		3.60	1.31, 9.92			2.41	0.69, 8.38	
pT4	4 (5.3%)		0.88	0.11, 7.29			0.73	0.07, 7.63	
pN		74			**<0.001**	74			0.5
pN0	44 (59%)		—	—			—	—	
pN1	15 (20%)		4.32	2.05, 9.12			1.82	0.58, 5.77	
pN2	15 (20%)		3.64	1.66, 8.01			1.19	0.30, 4.69	
Pn		75			0.5				
Pn0	72 (96%)		—	—					
Pn1	3 (4.0%)		1.69	0.41, 7.01					
L		75			**<0.001**	74			0.061
L0	49 (65%)		—	—			—	—	
L1	26 (35%)		3.66	1.94, 6.87			3.05	0.92, 10.1	
V		75			0.10				
V0	68 (91%)		—	—					
V1	7 (9.3%)		2.23	0.93, 5.35					
Grading		75			0.3				
G2	37 (49%)		—	—					
G3	38 (51%)		1.35	0.73, 2.53					
Residual Disease		75			**0.003**	74			**0.032**
R0	70 (93%)		—	—			—	—	
R1	5 (6.7%)		6.17	2.31, 16.5			3.93	1.26, 12.2	

Uni- and Multivariable Analysis – CD73 Adenocarcinoma									
	**Absolute**	**Univariable**				**Multivariable**			
**Characteristic**	**N = 75**[1]	**N**	**HR**[2]	**95% CI**[2]	**p-value**	**N**	**HR**[2]	**95% CI**[2]	**p-value**
Pleural Infiltration	28 (37%)	75			0.13				
No			—	—					
Yes			1.63	0.87, 3.04					
Metastatic Lymphnodes	0.00 (0.00, 2.00)	74	1.15	1.08, 1.24	**<0.001**				
Tumor Size in cm		75	1.15	1.00, 1.34	0.066	74	1.00	0.80, 1.25	>0.9
Neoadjuvant Therapy		75			0.3				
No			—	—					
Yes			1.77	0.69, 4.54					
Pack Years		32	1.01	0.99, 1.03	0.2				
SUVmax		74	1.00	0.99, 1.01	>0.9				

[1]n (%); Median (Q1, Q3)

[2]HR = Hazard Ratio, CI = Confidence Interval
